# Draft Genome Sequences of Fructobacillus fructosus DPC 7238 and Leuconostoc mesenteroides DPC 7261, Mannitol-Producing Organisms Isolated from Fructose-Rich Honeybee-Resident Flowers on an Irish Farm

**DOI:** 10.1128/MRA.01297-20

**Published:** 2020-12-10

**Authors:** Pradip V. Behare, Syed Azmal Ali, Olivia McAuliffe

**Affiliations:** a National Collection of Dairy Cultures Laboratory, Dairy Microbiology Division, ICAR-National Dairy Research Institute, Karnal, Haryana, India; b Proteomics and Cell Biology Laboratory, Animal Biotechnology Center, ICAR-National Dairy Research Institute, Karnal, Haryana, India; c Teagasc Food Research Centre, Fermoy, Cork, Ireland; d VistaMilk SFI Research Centre, Fermoy, Cork, Ireland; University of Delaware

## Abstract

Certain bacterial species, including some fructophilic lactic acid bacteria, are known to naturally produce the sugar alcohol mannitol. Here, we announce the draft genome sequences of the mannitol-producing organisms Fructobacillus fructosus DPC 7238 and Leuconostoc mesenteroides DPC 7261, which were isolated from fructose-rich honeybee-resident flowers found on an Irish farm.

## ANNOUNCEMENT

Fructophilic lactic acid bacteria (FLAB), which are present on fructose-rich fruits, flowers, and vegetables, are capable of tolerating high concentrations of fructose (1–3). Certain FLAB produce sugar alcohols, such as mannitol, which has attracted interest as a sugar substitute for diabetics and those with sugar intolerance due to its low calorie content (4). We previously isolated Fructobacillus fructosus DPC 7238 and Leuconostoc mesenteroides DPC 7261 from fructose-rich honeybee-resident flowers. Both strains display fructose utilization, with high mannitol yield (5). These strains have revealed their potential as application-specific starters in the development of innovative dairy products naturally sweetened with this low-calorie sugar (5).

F. fructosus DPC 7238 and L. mesenteroides DPC 7261 were grown overnight at 30°C in MRS broth (Becton, Dickinson and Co., Wokingham, Berkshire, UK) containing 10 g/liter fructose. Genomic DNA was extracted using the UltraClean microbial DNA isolation kit (MO BIO Laboratories, Cambridge, UK) and purified with the Isolate II PCR and gel kit (Bioline, Dublin, Ireland) according to the manufacturers’ instructions. Genomic DNA libraries were prepared using a Nextera XT library preparation kit (Illumina, San Diego, CA) and following the manufacturer’s protocol, with the following modifications: 2 ng of DNA instead of 1 ng was used as the input, and the PCR elongation time was increased from 30 s to 1 min. Libraries were sequenced on the Illumina HiSeq platform using a 250-bp paired-end read protocol (MicrobesNG, University of Birmingham, Birmingham, UK). Read quality was assessed using FastQC v0.11.7 (6). *De novo* assembly was performed with KmerGenie v1.6982 (7), Velvet v1.2.10 (8), SSPACE v3.0 (9), and GapFiller v1-10 (10). Annotation was performed using the NCBI Prokaryotic Genome Annotation Pipeline (PGAP) v4.3 (11). The final draft genomes were estimated to be ≥95% complete with ≤3% contamination using CheckM v1.0.12 (12). Default settings were used for all software.

The sequencing data statistics are shown in Table 1. The total numbers of coding genes and protein-coding regions in F. fructosus DPC 7238 are 1,444 and 1,368, respectively. Multiple RNAs were identified, i.e., 6 rRNA types, 49 tRNAs, and 3 noncoding RNAs. In the case of L. mesenteroides DPC 7261, 3,406 total coding genes and 3,229 protein-coding genes were identified, with 120 RNA regions including 13 rRNA types, 5 5S rRNAs, 101 tRNAs, and 6 noncoding RNAs. The numbers of pseudogenes (18 pseudogenes) and non-protein-coding sequences (18 sequences) determined for F. fructosus DPC 7238 are comparatively lower than those in L. mesenteroides DPC 7261 (57 each).

**TABLE 1:** Genomic features of the FLAB strains used in this study

Organism	Draft genome size (Mb)	No. of contigs	*N*_50_ (bp)	G+C content (%)	Mean coverage (×)	Total no. of reads	SRA accession no.	GenBank accession no.
F. fructosus DPC 7238	1.34	28	155,038	44.68	30	1,380,363	SRR12976643	JACTNH000000000
L. mesenteroides DPC 7261	3.24	56	157,699	40.34	30	3,343,285	SRR12976642	JACXBX000000000

Detailed analysis of the draft genome sequences of these fructose-tolerating, mannitol-producing organisms will shed further light on their ability to adapt to a fructose-rich environment and to produce mannitol from this substrate. These strains have the potential to be used as starter cultures or adjunct cultures for the manufacture of mannitol-enriched fermented dairy products and beverages.

### Data availability.

The draft whole-genome shotgun projects were deposited in DDBJ/ENA/GenBank. The SRA and GenBank accession numbers for F. fructosus DPC 7238 and L. mesenteroides DPC 7261 are listed in Table 1.
